# Minimally invasive cardiopulmonary bypass: does it really change the outcome?

**DOI:** 10.1186/cc5777

**Published:** 2007-04-15

**Authors:** Marco Ranucci, Giuseppe Isgrò

**Affiliations:** 1 Department of Cardiovascular Anesthesia and Intensive Care, IRCCS Policlinico S. Donato, Via Morandi 30, San Donato Milanese (Milan) – 20097, Italy

## Abstract

**Introduction:**

Many innovative cardiopulmonary bypass (CPB) systems have recently been proposed by the industry. With few differences, they all share a philosophy based on priming volume reduction, closed circuit with separation of the surgical field suction, centrifugal pump, and biocompatible circuit and oxygenator. These minimally invasive CPB (MICPB) systems are intended to limit the deleterious effects of a conventional CPB. However, no evidence exists with respect to their effectiveness in improving the postoperative outcome in a large population of patients. This study aimed to verify the clinical impact of an MICPB in a large population of patients undergoing coronary artery revascularization.

**Methods:**

We conducted a retrospective analysis of 1,663 patients treated with an MICPB. The control group (conventional CPB) was extracted from a series of 2,877 patients according to a propensity score analysis.

**Results:**

Patients receiving an MICPB had a shorter intensive care unit (ICU) stay, had lower peak postoperative serum creatinine and bilirubin levels, and suffered less postoperative blood loss. Within a multivariable model, MICPB is independently associated with lower rates of atrial fibrillation (odds ratio [OR] 0.83, 95% confidence interval [CI] 0.69 to 0.99) and ventricular arrhythmias (OR 0.45, 95% CI 0.28 to 0.73) and with higher rates of early discharge from the ICU (OR 1.31, 95% CI 1.06 to 1.6) and from the hospital (OR 1.46, 95% CI 1.18 to 1.8). Hospital mortality did not differ between groups.

**Conclusion:**

MICPBs are associated with reduced morbidity. However, these results will need to be confirmed in a large, prospective, randomized, controlled trial.

## Introduction

During the last decade, many attempts have been made to reduce the deleterious effects of cardiopulmonary bypass (CPB) in cardiac operations. Basically, these attempts have focused on specific changes to the standard equipment and management, acting on the nature of the materials composing the CPB circuit and oxygenator, on the technical aspects of the pump and the circuit, and on the priming volume and the anticoagulation management.

Biocompatible materials of different types have been applied, but the results of this strategy are still debated. In a large, multicenter, prospective, randomized trial, no impact on postoperative outcome was found in low-risk patients undergoing elective coronary artery bypass graft (CABG) surgery [1]. However, a similar study, which focused on high-risk patients, demonstrated a better outcome in patients treated with heparin-coated materials [2]. Reduction of systemic heparinization coupled with the use of biocompatible materials seems to limit some of the adverse effects of CPB [3-7]. New developments of coating systems for CPB materials have shown promising results [8-10]. Many authors [10-12] have stressed the negative action of open circuits (namely, of the reinfusion of shed blood from the pericardium) and proposed the use of closed circuits with separation of the surgical field suction blood. Finally, the need for limiting the circuit size in order to reduce the hemodilution degree has been thoroughly addressed in recent years, and many authors [13-17] have demonstrated the deleterious effects of severe hemodilution during CPB in terms of postoperative morbidity and mortality.

In response to the considerable amount of literature stressing the possible impact of CPB technology on postoperative outcome, the industry proposed new 'minimally invasive' CPB (MICPB) systems. Despite differences in biocompatible treatment used and other minor technical issues, these systems all have some points in common: they (a) are closed, (b) are treated with biocompatible coatings, (c) can be primed with a reduced fluid volume, (d) include a centrifugal pump, and (e) are equipped with systems for separation of the shed blood from the circuit.

However, despite enthusiastic reports on the limited number of patients treated with these MICPB systems [18-21], there is no clear information about the real impact of these technologies on the postoperative outcome of patients undergoing a cardiac operation. The present study aimed to determine the effects of an MICPB strategy on the postoperative outcome of a large population of patients undergoing CABG surgery.

## Materials and methods

### Study design

We conducted a retrospective study based on the Institutional Database for Cardiac Surgery including all patients undergoing CABG surgery at our institution from 1 January 2001 through 31 March 2006. The local ethical committee waived the need for approval, and all patients gave written consent to the scientific treatment of their data.

During the study period, we applied two distinct types of CPB management. Some patients received our conventional treatment based on a standard open circuit with roller or centrifugal pumps, conventional anticoagulation management, no biocompatible treatment, and no separation of the pericardial shed blood suction. These patients were considered the conventional CPB group. Other patients received an MICPB based on a closed circuit with separation of the pericardial shed blood suction, a centrifugal pump, phosphorylcholine-treated materials, and a reduction of systemic heparinization. These patients comprised the MICPB group. During the study period, no specific selection was carried out in assigning patients to the control or the MICPB group, assignment to groups was based only on the availability of the different CPB circuits. The use and availability of MICPB circuit were homogeneous during the whole study period and without a time-related bias. Closed circuits have been used for the last 10 years by all the perfusionists in our institution. Therefore, no selection bias based on the experience of the operating room team was expected.

### Patient population

Four thousand five hundred and forty patients undergoing isolated CABG operations were admitted to the study. Two thousand eight hundred and seventy-seven comprised the conventional CPB group, and 1,663 the MICPB group. Applying a propensity score analysis (see Statistics), we extracted a control group of 1,663 patients from the conventional CPB group.

### Anesthesia, surgery, and CPB management

Premedication included atropine sulphate, prometazine, and fentanyl. Anesthesia was induced with an intravenous infusion of remifentanil and a midazolam bolus. Cisatracurium besylate was later administered to allow tracheal intubation. Subsequently, the anesthesia was maintained with a continuous infusion of remifentanil and midazolam.

CPB was established via a standard median sternotomy, aortic root cannulation, and single-cannula atrial cannulation for venous return. As requested by the surgeon, the lowest core body temperature during CPB varied from 32°C to 37°C. Antegrade intermittent cold crystalloid or cold blood cardioplegia was used according to the surgeon's preference. The following equipment and techniques were applied in the control and MICPB groups:

In the control group, an open circuit with a hard-shell reservoir receiving blood from the venous cannulation, an active venting from the aortic root, and all the surgical field suctions were directly sent to the venous reservoir. The circuit was primed with 700 ml of a gelatin solution (Eufusin; Medacta Italia, Milan, Italy) and 200 ml of trihydroxymethylaminomethane solution. Roller (Stöckert, part of Sorin Group Deutschland GmbH, München, Germany) or centrifugal (Medtronic, Inc., Minneapolis, MN, USA) pumps were used according to availability in the control group. The oxygenator was a hollow-fiber D 905 Avant (Dideco, part of Sorin Group Italia S.r.l, Mirandola, Italy). The pump flow was targeted between 2.0 and 2.4 liters per minute per square meter and the target mean arterial pressure was settled at 60 mm Hg.

Anticoagulation was established with an initial dose of 300 IU per kilogram of body weight of porcine intestinal heparin injected into a central venous line 10 minutes before the initiation of CPB and with a target activated clotting time (ACT) of 480 seconds. At the end of CPB, heparin was reversed by protamine chloride at a 1:1 ratio of the loading dose, regardless of the total heparin dosage.

In the MICPB group, a closed circuit with a collapsible venous reservoir receiving blood from the venous cannulation and from a gravity venting from the aortic root was applied, and suction blood from the surgical field was actively drained into a hard-shell reservoir and never readmitted to the systemic circulation unless processed with a cell saver. All CPB surfaces were treated with a biocompatible coating (phosphorylcholine). This system, which has been published by our group [9,10], is manufactured on request by Dideco and is known as 'intraoperative ECMO (extracorporeal membrane oxygenation).' Anticoagulation was established with a target ACT of 300 seconds. The heparin loading dose was settled using the Hepcon HMS (Medtronic, Inc.).

No difference between groups existed with respect to the pump flow and pressure policy, the oxygenator, the priming nature, and the protamine administration protocol. In both groups, a cell saver was used throughout the surgical procedure. Tranexamic acid (15 mg/kg before CPB and 15 mg/kg after protamine) was used in all patients.

### Data collection and definitions

Using the Institutional Database for Cardiac Surgery, which follows the guidelines of the National Societies of Cardiothoracic Surgery and Cardiothoracic Anesthesia, we collected and analyzed the following preoperative data: demographics (age in years, gender, weight in kilograms, and height in centimeters), preoperative cardiovascular profile (ejection fraction, recent [30 days] myocardial infarction, unstable angina, use of antiplatelet agents [salycilates or clopidogrel/ticlopidine], congestive heart failure, previous vascular surgery, previous cardiac surgery, and use of intra-aortic balloon pump [IABP]), presence of comorbidities (chronic renal failure, diabetes on medication, chronic obstructive pulmonary disease [COPD], and cerebrovascular accident), and laboratory assays (serum creatinine value in milligrams per deciliters, serum bilirubin value in milligrams per deciliters, and hematocrit [HCT] percentage).

Operative data comprised primary operating surgeon, emergency procedure, number of distal coronary anastomoses, CPB duration in minutes, aortic cross-clamping duration in minutes, priming volume in milliliters, type of cardioplegic solution (crystalloid versus blood), lowest temperature in degrees Celsius, and lowest HCT percentage on CPB, total heparin dose in international units, and total protamine dose in milligrams.

Outcome variables included time on mechanical ventilation in hours, intensive care unit (ICU) stay in days, postoperative hospital stay in days, number of patients extubated early (within six hours from arrival in the ICU), number of patients discharged early from the ICU (the day after the operation), number of patients discharged early from the hospital (within five days from the surgical operation), peak postoperative serum creatinine level in milligrams per deciliters, peak postoperative serum bilirubin level in milligrams per deciliters, postoperative bleeding in milliliters (during the first 12 hours from the arrival to the ICU), surgical revision rate, need for homologous blood transfusions, perioperative myocardial infarction rate (new Q waves plus enzymatic criteria), low cardiac output syndrome (treated with inotropes or IABP), atrial fibrillation rate (not pre-existing), presence of ventricular arrhythmias, acute renal failure (requiring renal replacement therapy), stroke, peripheral thromboembolism (lower limb ischemia diagnosed on clinical plus echo-Doppler examination), severe pulmonary dysfunction, cardiac arrest, sepsis, and hospital mortality rate.

Beginning in January 2004, a specific fast-track program aimed at early discharge of patients from the ICU was applied. This program includes specific criteria for extubation and discharge from the ICU, which have been detailed in a recently published article [22]. The presence of this program was therefore included within the possible bias factors considered in the propensity score analysis and in the multivariable analysis.

### Statistics

All data are expressed as mean ± standard deviation of the mean or as absolute number and percentage when appropriate. A *p *value of less than 0.05 was considered significant for all statistical tests. The statistical analysis was performed using SPSS 11.0 software (SPSS Inc., Chicago, IL, USA).

The composition of the control group was obtained by extracting 1,663 patients from the conventional CPB group according to the propensity score technique. The following approach was applied:

#### Step 1

The conventional CPB group and MICPB group were compared for significant differences in pre- and intra-operative variables. Variables directly related to the different techniques (priming volume, lowest HCT on CPB, and heparin and protamine doses) were excluded from the analysis. By means of appropriate statistical tests (Student *t *test for unpaired data and Pearson's χ^2 ^test), 11 variables were found to be significantly different between groups: age, male gender, weight, ejection fraction, recent myocardial infarction, serum creatinine level, serum bilirubin level, COPD, cerebrovascular accident, diabetes on medication, and lowest temperature on CPB. With the exception of serum creatinine level, all of the comorbidities and risk factors were more frequent in the MICPB group.

#### Step 2

The 11 variables were entered into a multivariable stepwise forward logistic regression, and the use of MICPB was the dependent variable. The final predictive model for MICPB use included eight independent variables: weight (*p *= 0.002), age (*p *< 0.001), recent myocardial infarction (*p *< 0.001), serum creatinine level (*p *< 0.001), serum bilirubin level (*p *= 0.009), cerebrovascular accident (*p *= 0.011), diabetes on medication (*p *< 0.001), and lowest temperature on CPB (*p *= 0.031). On the basis of the logistic regression equation, each patient received a score (range, 0 to 1) that represented the 'propensity score' for being treated with MICPB.

#### Step 3

Patients in the MICPB group were divided into quintiles according to their propensity score. The first quintile (propensity score, 0 to 0.2) included 59 patients, the second (0.21 to 0.40) 848 patients, the third (0.41 to 0.60) 495 patients, the fourth (0.61 to 0.80) 259 patients, and the fifth (0.81 to 1) 2 patients. According to this distribution, the same number of patients for each quintile was randomly extracted from the conventional CPB file, resulting in the control group. Homogeneity of the groups for pre- and intra-operative variables was checked using a Student *t *test for unpaired data (between means differences) and a Pearson's χ^2 ^test (between frequencies differences).

The distribution of primary operating surgeons was analyzed for homogeneity between groups. During the study period, 2,727 patients (82%) were operated on by five surgeons, and the remaining 599 by four other surgeons. We therefore considered six groups based on the operating surgeon. The distribution did not differ significantly between the groups. The number of patients treated with the fast-track protocol (from January 2004) was 665 in the MICPB group and 632 in the control group (not significantly different).

The outcome of the two groups was compared using a Student *t *test for unpaired data (between means differences) and a relative risk analysis (with 95% confidence intervals) for mortality and for each specific morbid event. A multivariable logistic regression analysis was applied to the main outcome variables significantly associated with MICPB use in order to investigate the role of MICPB as an independent predictor of outcome within a model including other explanatory variables.

## Results

The control group created through the propensity score technique was homogeneous with the MICPB group for all the preoperative variables and comorbidities (Table 1). The logistic Euroscore did not differ significantly between the control (7.2 ± 11.4) and the MICPB (7.5 ± 11.8) groups. Operative variables did not differ significantly between groups. Of course, as a result of the different CPB techniques, patients in the MICPB group had a significantly lower priming volume and consequently a significantly higher nadir HCT value on CPB. As a result of the different anticoagulation protocol, they received significantly less heparin and protamine.

**Table 1:** Homogeneity of the groups for pre- and intra-operative variables

Variable	Control group	MICPB group	*P *value
	*n *= 1,663	*n *= 1,663	

Age (years)	67.9 ± 9.5	67.9 ± 9.2	NS
Weight (kg)	73.5 ± 12	73.7 ± 14	NS
Height (cm)	167 ± 8.6	167 ± 9.8	NS
Ejection fraction	0.50 ± 0.11	0.50 ± 0.11	NS
Serum creatinine level (mg/dl)	1.31 ± 0.7	1.28 ± 0.8	NS
Serum bilirubin level (mg/dl)	0.63 ± 0.38	0.61 ± 0.36	NS
Hematocrit (percentage)	39.2 ± 4.6	39.2 ± 4.4	NS
Priming volume (ml)	874 ± 190	771 ± 134	0.001
CPB duration (minutes)	60.9 ± 25	59.8 ± 21	NS
Aortic cross-clamping time (minutes)	34.4 ± 13	34.6 ± 13	NS
Lowest hematocrit on CPB (percentage)	26.2 ± 3.9	27 ± 3.7	0.001
Lowest temperature on CPB (°C)	33 ± 1.6	32.9 ± 1.4	NS
Total heparin dose (IU)	26,733 ± 32,159	23,590 ± 30,421	0.004
Total protamine dose (mg)	221 ± 58	162 ± 58	0.001
Distal coronary anastomoses	3.26 ± 0.95	3.37 ± 0.98	NS
Gender male	1,311 (78%)	1,322 (79.5%)	NS
Recent myocardial infarction	96 (5.8%)	107 (6.4%)	NS
Unstable angina	240 (14.4%)	203 (12.2%)	NS
Antiplatelet agents	350 (21%)	338 (20.3%)	NS
Previous vascular surgery	93 (5.6%)	93 (5.6%)	NS
Congestive heart failure	40 (2.4%)	38 (2.3%)	NS
Preoperative IABP use	10 (0.6%)	11 (0.6%)	NS
COPD	145 (8.7%)	151 (9.1%)	NS
Cerebrovascular accident	104 (6.2%)	91 (5.5%)	NS
Diabetes on medication	312 (18.7%)	344 (20.1%)	NS
Chronic renal failure	40 (2.4%)	38 (2.3%)	NS
Redo operation	45 (2.7%)	40 (2.4%)	NS
Emergency procedure	13 (0.8%)	9 (0.5%)	NS

CPB, cardiopulmonary bypass; COPD, chronic obstructive pulmonary disease; IABP, intra-aortic balloon pump; MICPB, minimally invasive cardiopulmonary bypass; NS, not significant.

In the univariate analysis, the postoperative outcome (Table 2) was affected by a higher morbidity in the control group. Patients in this group experienced more postoperative bleeding and had higher postoperative peak values of both serum creatinine and bilirubin. MICPB significantly reduced the risk for receiving an IABP and for atrial fibrillation, ventricular arrhythmias, cardiac arrest, and peripheral thromboembolism. Due to the reduced morbidity, patients treated with an MICPB had a significantly shorter ICU stay, and the rates of patients extubated early and discharged early from the ICU or the hospital were significantly higher (1.5 to 2 times) in the MICPB group. No significant difference was observed in terms of hospital mortality.

**Table 2:** Outcome variables in the two groups

Variable	Control group	MICPB group		*P *value
	*n *= 1,663	*n *= 1,663		

Mechanical ventilation time (hours)	20.2 ± 79	17.3 ± 38		NS
ICU stay (days)	2.9 ± 4.3	2.5 ± 3.1		0.001
Postoperative hospital stay (days)	8.4 ± 5.9	8.1 ± 6.2		NS
Peak serum creatinine level (mg/dl)	1.43 ± 0.7	1.24 ± 1		0.001
Peak serum bilirubin level (mg/dl)	1.07 ± 0.79	0.89 ± 0.71		0.001
Bleeding (ml/12 hours)	505 ± 396	458 ± 342		0.001

Variable	Count (percentage)	Count (percentage)	RR and 95% CI	*P *value

Early extubation	139 (8.3)	197 (11.8)	1.47 (1.17–1.85)	0.001
Early ICU discharge	307 (18.4)	556 (33.4)	2.21 (1.89–2.6)	0.001
Early hospital discharge	187 (11.2)	316 (19)	1.85 (1.52–2.25)	0.001
Homologous blood transfusions	751 (45.1)	764 (45.9)	1.03 (0.9–1.18)	NS
Surgical reoperation	79 (4.7)	64 (3.8)	0.8 (0.57–1.12)	NS
Myocardial infarction	58 (3.5)	47 (2.8)	0.8 (0.54–1.19)	NS
Major inotropic support	177 (10.6)	170 (10)	0.95 (0.76–1.19)	NS
Intra-aortic balloon pump	49 (2.9)	28 (1.7)	0.56 (0.35–0.9)	0.015
Atrial fibrillation	293 (17.6)	251 (15.1)	0.83 (0.69–0.99)	0.049
Ventricular arrhythmias	59 (3.5)	26 (1.5)	0.43 (0.27–0.69)	0.001
Cardiac arrest	19 (1.1)	3 (0.2)	0.15 (0.04–0.53)	0.001
Acute renal failure	39 (2.3)	38 (2.3)	0.97 (0.62–1.53)	NS
Stroke	10 (0.6)	14 (0.8)	1.4 (0.62–3.16)	NS
Peripheral thromboembolism	6 (0.4)	0 (0)	-	0.014
Severe lung dysfunction	22 (1.3)	14 (0.8)	0.63 (0.32–1.24)	NS
Sepsis	10 (0.6)	14 (0.8)	1.4 (0.62–3.16)	NS
Hospital mortality	55 (3.3)	50 (3)	0.9 (0.61–1.33)	NS

CI, confidence interval; ICU, intensive care unit; MICPB, minimally invasive cardiopulmonary bypass; NS, not significant; RR, relative risk.

The main outcome variables significantly associated with the use of an MICPB system were analyzed using a multivariable stepwise forward logistic regression analysis. After correction for the other explanatory variables, MICPB remained independently associated with a better outcome, with the exceptions of early extubation rate and postoperative IABP use (Table 3).

**Table 3:** Multivariable analysis (stepwise forward logistic regression) for MICPB impact on outcome variables

Outcome variable	OR (95% CI)	*P *value	Adjusted for
Early extubation	1.34 (0.68–1.12)	0.27	Age, recent MI, serum creatinine value, fast-track program
Early ICU discharge	1.31 (1.06–1.6)	0.001	Gender, ejection fraction, serum creatinine value, unstable angina, cerebrovascular accident, CPB duration, fast-track program
Early hospital discharge	1.46 (1.18–1.8)	0.001	Gender, recent MI, serum creatinine value, chronic obstructive pulmonary disease, CPB duration, lowest temperature on CPB, fast-track program
Postoperative intra-aortic balloon pump	0.7 (0.4–1.17)	0.17	Age, ejection fraction, recent MI, CPB duration, lowest hematocrit on CPB
Atrial fibrillation	0.83 (0.69–0.99)	0.049	Gender, ejection fraction, preoperative hematocrit
Ventricular arrhythmias	0.45 (0.28–0.73)	0.001	Ejection fraction, serum creatinine value
Cardiac arrest	0.15 (0.04–0.5)	0.002	Congestive heart failure

CI, confidence interval; CPB, cardiopulmonary bypass; ICU, intensive care unit; MI, myocardial infarction; MICPB, minimally invasive cardiopulmonary bypass; OR, odds ratio.

## Discussion

Since in the mid-1980s, many attempts to improve the quality of CPB have been pursued. This trend toward a CPB improvement, which led to the introduction of hollow-fiber oxygenators, centrifugal pumps, and biocompatible treatments of the circuit and oxygenator decreased considerably in the early 1990s due to the emerging off-pump coronary surgery. In recent years, the new challenge has been to improve CPB quality to the point of making this technique competitive even with off-pump coronary surgery. Not by chance, during the last five or six years, the market has been offering an overwhelming number of new products (non-heparin-based biocompatible treatments, new generation centrifugal pumps, and MICPB equipment) aimed at improving the quality of CPB.

At present, at least three major companies are proposing an 'MICPB system.' With the exception of a few differences related mainly to safety devices (aimed at detecting and eliminating air entering the circuit), they share the same philosophy: closed circuit, centrifugal pump, hollow-fiber oxygenator, biocompatible surfaces, separation of the pericardial shed blood suction, and reduced priming volume. Biocompatible treatments of the circuit and oxygenator currently available on the market differ in nature (heparin, phosphorylcholine, sulphate-sulphonate groups, and so on) but exert a well-established action in limiting thrombin formation, platelet-count decrease, and inflammatory reaction [3,5,8,9]. On a practical basis, commercially available integrated MICPB systems require a specific expertise, have a prolonged learning curve, need team work, and are more expensive than conventional circuits. Moreover, some concerns with respect to their safety have been raised [23]. Therefore, their use is justified only if their positive impact on postoperative outcome is well proven.

Unfortunately, this point is far from being clarified. Scientific reports on the use of MICPB systems are limited, represented mainly by case reports [20] or prospective trials enrolling limited numbers of patients [18,19]. The general feeling is good, and various advantages have been reported: shorter ICU stay, decreased need for inotropes, less myocardial injury, lower rate of atrial fibrillation [18], and blunting of the inflammatory reaction and of activation of the coagulation and fibrinolytic systems [19]. The main risk with these initial trials on new therapeutic or technical systems is that patients in the study group may receive better care and report improved outcomes merely because they are being studied and received a new treatment; this bias is quite common and is known as the Hawthorne effect [24]. In a series of articles, Remadi and colleagues [25-27] analyzed their experience with one of the commercially available systems. In the first article [25], they basically address the safety issues, practical feasibility, and general outcome of patients treated with an MICPB system. Subsequently, they published a prospective randomized trial on 100 patients undergoing aortic valve surgery [26], where patients treated with the MICPB had less neurologic events and a better preservation of renal function but no differences in mortality. Finally, they recently published a prospective randomized trial on 400 CABG patients [27], where the MICPB group demonstrated a lower rate of low cardiac output syndromes, need for allogeneic blood transfusions, and a better preservation of the renal function (lower peak values of creatinine and urea); mortality was not significantly different between groups.

The present study represents by far the largest experience with MICPB in CABG operations. The main strength of this study is the patient population size: more than 3,200 patients were analyzed. Conversely, the main limitation is that it is not prospective and randomized, the control group having been retrospectively created using a propensity score matching.

The main results of our study are that MICPB does not modify mortality but exerts a considerable beneficial effect on postoperative morbidity. In this respect, our data are in agreement with those of Remadi and colleagues [26,27], who could not demonstrate different mortality rates in their prospective randomized trials. In our study, myocardial function was not addressed with specific markers; we saw a lower rate of patients receiving IABP support in the MICPB group in the univariate analysis, but this difference lost significance when corrected for confounding factors. Other articles suggest a better myocardial protection exerted by MICPB [18,27] and a better hemodynamic profile [28], but we cannot confirm this on the basis of our data. However, there are lower rates of atrial fibrillation, ventricular arrhythmias, and cardiac arrest. Atrial fibrillation after cardiac surgery may recognize an inflammatory triggering mechanism [29-31], and there is evidence of blunting of the inflammatory reaction in patients treated with MICPB [19] or when biocompatible circuits and separation of the shed blood suction are applied [32]. We therefore are inclined to attribute the atrial fibrillation containment to a better control of the inflammatory reaction in MICPB patients, but we recognize that we have no information about the possible role of prophylactic strategies (that is, perioperative beta-blocker use) within our study group.

In agreement with other studies [26,27], we can confirm that MICPB systems exert a protective effect on perioperative renal function: the postoperative increase of serum creatinine was lower in the MICPB group even if the rate of patients requiring a dialytic treatment was not significantly different. Moreover, the peak postoperative serum bilirubin is lower in MICPB patients. Our MICPB has at least two characteristics that can justify a beneficial effect on the renal side: it guarantees higher HCT values during CPB and is equipped with biocompatible surfaces. Limiting hemodilution during CPB decreases postoperative renal dysfunction rate [13-17], and biocompatible surfaces have been associated with a reduced rate of renal dysfunction in high-risk patients [2].

Peripheral thromboembolic events are less frequent when the MICPB is used. This can be attributed to the lower coagulation system activation, thrombin generation [32], and the antithrombin-saving effect already demonstrated with this technique [7]. However, we could not demonstrate that the overall rate of thromboembolic events (including myocardial infarction, stroke, pulmonary embolism, and mesenteric infarction) was lower in the MICPB group.

In our series, MICPB is associated with decreased postoperative bleeding but not with a lower transfusion rate. This finding is somewhat surprising, contradicting other studies [27] and our own finding in a published study on a limited number of patients [10]. However, we lack important information about the amount of blood product administered and cannot rule out that patients in the MICPB group may have received less blood product. Moreover, the lower postoperative bleeding detected is significant but clinically trivial (50 ml in 12 hours) and therefore unlikely to determine differences in transfusion rate. Finally, as a result of this better multifactorial outcome, patients in the MICPB group had a shorter ICU stay and higher rates of early ICU and hospital discharge.

Our MICPB system has some differences with respect to other commercially available products. It is not preassembled, and the venous return is not actively drained by a centrifugal pump, but passively gravity-drained into a collapsible reservoir; however, this difference does not result in a higher priming volume. In this respect, it is similar to the one proposed by Aldea and colleagues [5,6,32]. In their experience, this group demonstrated that a closed, heparin-bonded circuit with a reduction of systemic heparinization exerted a beneficial effect on postoperative outcome [5], reducing blood loss and transfusions, shortening mechanical ventilation time and ICU and hospital stay, and reducing postoperative complications (namely, thromboembolic events). The main characteristic of our system is that, due to the presence of a collapsible venous reservoir, no specific expertise or prolonged learning curve is required of the perfusionist. Moreover, the risk of air entering the circuit is not different from that of conventional open circuits, and no specific safety devices are required. Conversely, the preassembled circuits available on the market are generally based on an active venous return with the inlet of the centrifugal pump directly connected to the venous line. This almost invariably leads to the risk of air entering the circuit. Therefore, these circuits are equipped with bubble detectors and automated clamps allowing air to be eliminated from the circuit. Of course, this complex extracorporeal circuit needs a specific expertise, and doubts about the safety of the technique have been raised [23].

Heparin dose reduction associated with MICPB is still an open question. In the majority of studies published, the anticoagulation protocol is not changed [18-21,25-27], whereas in our study MICPB was associated with a reduced heparin dose. Aldea and colleagues [5,6] demonstrated that this approach is associated with a better outcome and does not induce adverse events. Øvrum and colleagues [4], in an impressive series of 5,658 patients, demonstrated that a strategy of reduced heparinization plus heparin-bonded circuits led to a postoperative outcome probably better than what was reported in the literature for off-pump CABG patients.

We do not think that heparin dose reduction *per se *is responsible for the better outcome of our MICPB patients. Simply, because the thrombin generation with MICPB appears to be reduced, less heparin is probably needed. Moreover, it is probably not useful to speculate which one of the single aspects included in an MICPB strategy (closed circuit, biocompatible surfaces, reduced hemodilution, heparin dose reduction, and so on) is responsible for the better outcomes. MICPB should be considered a multifactorial strategy, aimed to counteract the multifactorial deleterious effects of a conventional CPB. Within this model, further improvements to the MICPB can be considered (for example, the use of specific soft-flow arterial cannulas to reduce the traumatic effect exerted by high-velocity blood flow on the aortic wall). However, due to the absence of active venting, the system that we are using at present is inadequate for non-isolated CABG operations and for valvular procedures. A possible improvement to the system is the use of a closed venous reservoir actively draining the systemic venous blood (by means of an external negative pressure) which could be used for active venting of the left-sided cardiac cavities.

With respect to previous studies that failed to demonstrate a beneficial effect of heparin-coated, closed circuits, the main difference was the use of a complete MICPB system, including separation of the suction from the surgical field and a reduced priming volume.

## Conclusion

The use of MICPB in coronary patients undergoing surgical revascularization is associated with an improvement in postoperative outcome. We did not find any difference in mortality, but given that this figure was approximately 3%, the study was probably underpowered in this respect. Even though our population was large and the selection bias was reduced by the propensity score analysis, our results will need to be confirmed in a large, prospective, randomized, controlled trial.

## Key messages

• An MICPB system is based on the simultaneous use of closed circuits, biocompatible surface treatment, low priming volume, and separation of the suction from the surgical field.

• The use of a collapsible venous reservoir limits the need for specific expertise in using these systems.

• The use of an MICPB system in coronary operations is associated with decreased postoperative morbidity and with earlier ICU and hospital discharge.

## Abbreviations

ACT = activated clotting time; CABG = coronary artery bypass graft; COPD = chronic obstructive pulmonary disease; CPB = cardiopulmonary bypass; HCT = hematocrit; IABP = intra-aortic balloon pump; ICU = intensive care unit; MICPB = minimally invasive cardiopulmonary bypass.

## Competing interests

The authors declare that they have no competing interests.

## Authors' contributions

MR contributed to the conception and design of the study and to the statistical analysis and gave the final approval of the manuscript. GI contributed to the acquisition of data and to the statistical analysis. Both authors had full access to the data and take responsibility for its integrity and read and approved the final manuscript.
